# Indigestible Food

**Published:** 1886-01

**Authors:** 


**Indigestible Food.**—In estimating the value of food, it is not alone sufficient to take into account its nutritive elements. A substance may contain every desirable element of food, or some particularly valuable one, and yet be totally unfit for eating on account of its inherent indigestibility, or the tendencies of the digestive organs of the eater. That which may be eaten with satisfaction and impunity by one person may be nauseous and injurious to another. Experience proves that it is better for some persons, in extreme cases, to become vegetarians, and for others to take a large proportion of animal food. Indigestibility may be broadly alleged against everything unripe, and against most things uncooked, though some persons can eat raw, or even unripe fruit, with impunity, enough to cause painful illness to others.

